# Untangling the Copy Number Variation at the Basis of Belted Phenotypes in Cattle Using Long‐Read Sequencing

**DOI:** 10.1002/age.70159

**Published:** 2026-07-17

**Authors:** Rensco A. H. Hogers, Mirte Bosse, Annemieke P. Rattink, Rayner González‐Prendes, Marta Gòdia, Kimberley Laport, Martien A. M. Groenen, Richard P. M. A. Crooijmans, Aniek C. Bouwman

**Affiliations:** ^1^ Animal Breeding and Genomics Wageningen University & Research Gelderland the Netherlands; ^2^ Amsterdam Institute of Life and Environment (A‐Life) VU University Amsterdam Noord‐Holland the Netherlands; ^3^ Centrum voor Genetische Bronnen Nederland Wageningen University & Research Gelderland the Netherlands

**Keywords:** belted phenotype, cattle, copy number variation (CNV), long‐read sequencing, selective sweep, *TWIST2*

## Abstract

The belted phenotype in cattle, a distinctive white belt across the midsection observed in breeds such as the Dutch Belted and Belted Galloway, is caused by a copy number variant (CNV) upstream of *TWIST2*, a gene involved in melanocyte development. While the CNV is necessary for belt formation, the genetic basis of variation in belt width and completeness remains unclear. To refine understanding of these phenotypes, we generated a *de novo* genome assembly of Dutch Belted cattle and analysed both short‐ and long‐read sequencing data from animals with normal‐width, small‐width, and incomplete belts. Standard variant calling tools failed to identify the CNV, highlighting technical challenges in detecting large insertions. Instead, coverage analysis and qPCR confirmed the CNV as a 6 kb tandem array approximately 16.5 kb upstream of *TWIST2*. Long‐read evidence showed identical four‐copy haplotypes for individuals with normal‐width, small‐width, and incomplete belts, indicating that CNV copy number does not influence belt width or completeness. Furthermore, no unique variants were detected within the CNV or *TWIST2* in a small‐width belted individual, suggesting that modifiers affecting belt width likely reside elsewhere in the genome. Genome‐wide scans revealed extremely low nucleotide diversity in the CNV region, consistent with a selective sweep driven by strong artificial selection unique to the Dutch Belted breed. Together, these results show that while the CNV is necessary for belt formation, additional modifier loci likely influence belt width and completeness.

## 1 Introduction

Cattle are home to many different coat colours and patterns, but one of the most striking is the belted phenotype. This trait is characterised as a white band encircling the midsection of an animal and varies in location, width, and completeness. The belted phenotype is not unique to cattle; it also occurs in other species, such as mice, pigs, and goats, although its appearance differs both across and within species. In mice, it features as a narrow belt close to the hindlimbs (Murray and Snell 1943), in pigs it is positioned more anteriorly (Porter 1993), and in cattle it spans the region between the fore‐ and hindlimbs (Kuiper Jr 1920). Given the variation among species, it is not surprising that distinct genetic mechanisms underlie the phenotype in different species. The causal mutation in mice was detected within the gene *adamts20* (Rao et al. 2003), while in pigs the trait is associated with variants in the *KIT*, *EDNRB*, and *MC1R* genes (Giovannini et al. 2023; Giuffra et al. 1999). In cattle the phenotype is caused by a copy number variant (CNV) upstream of the *TWIST2* gene (Awasthi Mishra et al. 2017). Despite involving different genes across species, all of these loci influence melanocyte development, migration, or pigment production, indicating convergence on the same overarching pigmentation pathway (Kelsh et al. 2009; Nasti and Timares 2015; Saldana‐Caboverde and Kos 2010; Silver et al. 2008; Yoshida et al. 2001).

Several cattle breeds carry the belted phenotype, including the Dutch Belted, Brown Swiss, and Belted Galloway, all with varying degrees of focus on the completeness and width of the belt. The Dutch Belted Studbook is especially strict, as the belt must not only be present, but also complete (fully encircling the midsection) with a width of at least 25 cm (Runderen 2021). However, despite these strict requirements, aberrations, such as incomplete or narrow belts, are still found. Over a century ago, breeders already noted the challenge of selecting for the pattern, as doing so reduced overall conformation and productivity (Kuiper 1921). Furthermore, a lowered interest in this breed as a production animal has caused a reduction in the effective population size, which, in combination with a breeding focus on the belted phenotype, has resulted in high levels of inbreeding, with rates between 0.25% and 0.50% per generation for the Dutch Belted (Centrum voor Genetische bronnen 2024). Due to these factors, the population is currently considered “at risk” (Food and of the United Nations, A.O 2023). The strong selection pressure on the belt, combined with the reduced effective population size, suggests that genomic regions underpinning the trait may show reduced genetic diversity or signatures of selective sweeps.

Against this backdrop, researchers have steadily unravelled the genetics of the belted phenotype in cattle. The belted trait was found to be dominant (Kuiper 1921), and refined by Schmutz et al. (2001) to be monogenic autosomal dominant, though homozygosity does not guarantee a complete belt. The underlying molecular mechanism that causes the belt also become known, namely due to a lack of melanocytes in the white part of the belt (Drögemüller et al. 2009). Initial linkage mapping of the belted locus in Brown Swiss cattle localised the causal variant to a 922 kb telomeric region of chromosome 3 (Drögemüller et al. 2009). Including Belted Galloway and Dutch Belted cattle narrowed this interval to 336 kb and suggested a shared founder across breeds (Drögemüller et al. 2010). Ultimately, the phenotype was linked to a 6 kb CNV located 16 kb upstream of *TWIST2* (Awasthi Mishra et al. 2017), later confirmed in European and Siberian populations (Rothammer et al. 2018). It has been shown that there is a considerable difference in the CNV copy number in the belted cattle population, ranging from only three copies to a maximum of 12 copies, whereas non‐belted individuals only have two (Awasthi Mishra et al. 2017; Rothammer et al. 2018). *TWIST2* plays an important regulatory role during neural crest and melanocyte precursor development, and altered gene dosage or misexpression could plausibly influence the extent or timing of melanocyte migration, potentially contributing to variation in belt width or completeness (Awasthi Mishra et al. 2017; Gitelman 2007; Simões‐Costa and Bronner 2015). These earlier studies, however, relied primarily on short‐read sequencing, SNP‐chip intensities, and qPCR‐based estimate approaches that are often unable to resolve complex or repeat‐rich structural variants accurately (Sedlazeck et al. 2018). Furthermore, the past studies mainly focused on the presence/absence of the belt, and as a result, the true structure, haplotype composition, and possible functional variation within the CNV remain incompletely characterised.

This raises three unanswered questions: whether CNV copy number influences belt width and belt completeness, whether signatures of selection are evident around the CNV, and whether additional genetic modifiers outside the CNV, similar to secondary loci affecting belt width in pigs (Giuffra et al. 1999), contribute to the phenotype in cattle. To address these knowledge gaps, we generated a *de* *novo* genome assembly of Dutch Belted cattle and combined short‐ and long‐read sequencing in order to: refine the structure and haplotype architecture of the CNV, test whether CNV copy number correlates with belt width or completeness, search for additional variants unique to a small‐width belted individual, and assess nucleotide diversity around the CNV to identify possible signatures of selection.

## 2 Materials and Methods

### 2.1 Sample Collection

Sperm samples of ten Dutch Belted bulls with normal‐width belts were retrieved from the Centre for Genetic Resources, the Netherlands (CGN). Blood samples from an additional ten Dutch Friesian Red and nine Groningen White Headed individuals obtained from CGN were included for comparative nucleotide diversity analyses. Blood from five belted cows (one with a normal‐width belt, one with a small‐width belt, and three with an incomplete belt) was collected during routine health inspection and included in the study. For qPCR‐based copy number analysis, hair samples were collected from six Dutch Belted animals with atypical belt phenotypes (three small‐width belted and three incomplete‐belted) and one non‐belted control individual.

### 2.2 DNA Extraction and Sequencing

Short‐read sequencing of the sperm‐derived samples was performed using the Illumina HiSeq platform (Illumina Inc., USA) at 10× coverage with paired‐end 150 bp reads. For the small‐width and incomplete belted Dutch Belted cows, DNA was extracted from blood using the NucleoSpin Tissue Kit (BIOKÉ) with Proteinase K. Libraries were prepared with the Illumina DNA PCR‐Free Prep kit (Illumina Inc., USA) and paired‐end sequenced (150 bp) on the Illumina NovaSeq6000 (Illumina Inc., USA). For long‐read sequencing of the same two cows, DNA was extracted with the GENTRA Blood kit (Qiagen N.V.). The quality and quantity of DNA were evaluated using 0.6% (w/v) agarose gel electrophoresis and a Qubit dsDNA Broad Range kit (Qiagen N.V.). DNA was first sheared using a g‐TUBE (Covaris, USA) centrifuged at 1200 g_*n*_, and then treated with Short Read Eliminator XS (Pacific Biosciences of California Inc., USA) to eliminate fragments under 10 kb. Libraries were prepared using the SQK‐LSK110 kit (Oxford Nanopore Technologies plc, UK) and sequenced on a PromethION with an R9.4.1 flow cell (Oxford Nanopore Technologies plc, UK). For the three incomplete‐belt cows, DNA was extracted and treated with SRE as described above. A pooled library was prepared from all three samples using the Native Barcoding Kit 24 V14 (SQK‐NBD114.24). MinKNOW was supplied with the BTA3 reference FASTA from ARS‐UCD2.0 (RefSeq NC_037330.1) containing the CNV region of interest for adaptive sampling, and three loadings were performed on a single PromethION Flow Cell (R10.4.1).

### 2.3 CNV Detection

To identify the CNV in the long‐ and short‐read data, we aligned all the normal‐width Dutch Belted samples to ARS‐UCD2.0 (GenBank Bioproject PRJNA391427), using Minimap2 v2.28‐r1209 (Li 2018). For short‐read data the ‐ax sr preset for Minimap2 was used, while for the long‐read data the ‐ax map‐ont preset was used. For individuals with multiple sequencing runs, alignment files were merged with Samtools merge v1.20 (Danecek et al. 2021), followed by sorting and indexing with Samtools sort and Samtools index. Variant calling was carried out with multiple tools, as no single method is capable of detecting all types of genome variants and a mixture of callers improves the likelihood of recovering the full spectrum of present variants (Aydin et al. 2025; Joe et al. 2024). We used smoove v0.2.8 (https://github.com/brentp/smoove) in combination with duphold v0.2.3 (Pedersen and Quinlan 2019), delly v1.2.6 (Rausch et al. 2012), CNVnator 0.4.1 (Abyzov et al. 2011), and sniffles2 v2.8.0 (Smolka et al. 2024). No additional BAM‐level quality filters were applied beyond those implicit in each tool's default settings, nor were results harmonised across callers; this was intentional, as the aim was to evaluate whether the CNV could be detected in a standard, unoptimised analysis workflow without prior knowledge of the variant. Smoove, Delly, and CNVnator were applied to the short‐reads, while sniffles2 was used with the long‐reads. Smoove, Delly, and sniffles2 were run with default parameters, CNVnator, was executed with default parameters but with three bin sizes (1000, 6500, and 10 000) to evaluate performance across different resolution scales.

To refine the CNV boundaries, we combined computational and visual approaches. Nucleotide BLAST (Altschul et al. 1990) was run with the forward primer described by (Awasthi Mishra et al. 2017) against ARS‐UCD2.0. The resulting region was further inspected in Jbrowse v1.16.11 (Buels et al. 2016) using long‐read alignments, in order to identify structural breakpoints consistent with CNV boundaries.

### 2.4 *de Novo* Assembly

To gain insights into the CNV, and to compare a Dutch Belted animal with a small belt to one with a normal‐width belt, we constructed a *de novo* genome assembly of a Dutch Belted individual with a normal‐width belt. Long‐reads from the normal‐width belt individual were first converted from FAST5 to POD5 using the POD5 file format v0.2.3 (https://github.com/nanoporetech/pod5‐file‐format). Basecalling was performed using Guppy v6.5.7 with the dna_r9.4.1_450bps_sup.cfg configuration file. Reads were filtered with Filtlong v0.2.1 (https://github.com/rrwick/Filtlong), removing sequences shorter than 1000 bp and the 10% lowest‐quality. Subsequently, the ONT reads were trimmed using Porechop_ABI v0.5.0 (Bonenfant et al. 2022), using the *ab initio* mode. The trimmed reads were *de novo* assembled using Flye v2.9.3 (Kolmogorov et al. 2019). Polishing was carried out using both the ONT long‐reads and Illumina short‐reads (trimmed using trimmomatic v0.39 (https://github.com/usadellab/Trimmomatic) with NextPolish v1.4.1 (Hu et al. 2020)). The assembly was then scaffolded to ARS‐UCD2.0 with RagTag v2.1.0 (Alonge et al. 2022).

To investigate repeat content within the CNV, RepeatModeler (v2.0.5) (Flynn et al. 2020) with LTR structural analysis enabled was used to find repeats in the *de novo* assembly. Subsequently, RepeatMasker (v4.1.6) (Smit et al. 2013–2015) was used with this custom repeat library to soft mask the assembly. Both tools were run in the Dfam TE Tools Container (https://github.com/Dfam‐consortium/TETools).

Assembly and read quality were evaluated using BUSCO v5.6.1 with the cetartiodactyla_odb10 dataset (Manni et al. 2021), QUAST v5.2.0 (Mikheenko et al. 2018), pycoQC v2.5.2 (Leger and Leonardi 2019), and FastQC v0.12.1; https://www.bioinformatics.babraham.ac.uk/projects/fastqc/. Results were summarised with MultiQC v1.19 (Ewels et al. 2016). BUSCO and QUAST were used to assess the quality of the finished assembly, pycoQC was used to analyse the quality of the raw ONT long‐reads, and FastQC was used to assess the quality of the trimmed long‐ and short‐reads. All steps were implemented in a Snakemake v7.32.4 (Köster and Rahmann 2012) pipeline, available on GitHub: https://github.com/RenscoHogers/Bovine‐Assembly‐Pipeline.

### 2.5 CNV Copy Number Quantification

Hair‐derived DNA from the six atypical‐phenotype animals and the non‐belted control (see Sample Collection) was analysed using qPCR to determine CNV copy number. The primers were designed with Primer3 (https://github.com/primer3‐org/primer3) using BTA3:118 029 001–118 034 401 as the CNV location in ARS‐UCD1.2 (GenBank Bioproject PRJNA391427), resulting in the following set of primers: CNV_forward_primer: 5′‐CCTGTCCATCACCAACTCCT‐3′; CNV_reverse_primer: 5′‐GGGGATGACAGAGGATGAGA‐3′. qPCR reactions were performed in triplicate on a QuantStudio 5 system (Thermo Fisher Scientific Inc., USA). The final reaction (12.5 μL) included 1.25 μL (2 μM) of each primer and 3.7 μL of DNA (10 ng/μL) diluted in 1:3.6 and 6.25 μL 2× reaction buffer MESA BLUE qPCR MasterMix Plus for SYBR Assay Low ROX (Eurogentec, Belgium) on a Quantstudio 5 system from Applied Biosystems (Thermo Fisher Scientific Inc., USA) using the Comparative Ct (delta Ct) method. Ct values from triplicate wells were averaged. $\Delta \Delta C_{t}$ was calculated as the difference between each sample's Ct and the average Ct of non‐belted controls (copy number = 2). Copy number was inferred as $2\times 2^{-\Delta \Delta C_{t}}$. The Thermal profile was: 50°C for 2 min, 95°C for 10 min, and 40 cycles at 95°C for 15 s and 60°C for 1 min, followed by a melting curve stage (95°C for 15 s, 60°C for 1 min and 95°C for 15 s).

For the samples not analysed by qPCR, copy number was estimated from short‐read sequencing data using a coverage ratio approach. Mean sequencing coverage within the CNV region was calculated with Mosdepth (v0.3.8) (Pedersen and Quinlan 2018) and normalised to the mean coverage across the entirety of BTA3. Copy number (CN) was then estimated as $\mathrm{CN}=\left({\text{Coverage}}_{\mathrm{CNV}}/{\text{Coverage}}_{\mathrm{BTA}3}\right)\times 2$, assuming a diploid baseline of two copies for BTA3. Additionally, long‐read sequencing data were used to characterise CNV structure: reads spanning the CNV region were extracted, aligned against the ARS‐UCD2.0 reference using BLAST (Altschul et al. 1990), and manually inspected to count individual CNV copies and confirm their arrangement relative to the reference.

### 2.6 Belt‐Width Variant Discovery

To identify potential genetic elements that may influence the width of the belted phenotype, we compared 10 Dutch Belted individuals with normal‐width belts to one small‐width belted individual. Genomic variants were detected in the small‐width belted sample using Project nf‐EXPLOR v0.0.2 (https://github.com/tuannguyen8390/nf‐EXPLOR, which performed quality control, SNP calling, and structural variant (SV) calling). Quality control of both long‐ and short‐reads was performed using FiltLong v0.2.2 with the parameters ‐‐min_length 200, ‐‐trim, ‐‐split 1000, and ‐‐min_mean_q 90. Reads were mapped against the *de novo* generated Dutch Belted genome using Minimap2 v2.24 with the ‐ax map‐ont preset. SNP calling was performed using Clair3 v1.0.5 with the r941_prom_sup_g5014 model, and SV calling was performed using Sniffles2 v2.2 with the ‐‐reference option. Reporting was done using NanoPlot (De Coster and Rademakers 2023), Mosdepth v0.0.3 (Pedersen and Quinlan 2018) and MultiQC v1.14 (Ewels et al. 2016).

The resulting SNPs in the small‐width belted sample were compared against a second SNP dataset obtained by variant calling the short‐reads of the 10 Dutch Belted samples to the *de novo* Dutch Belted genome assembly using Clair3 v1.0.9 with the Illumina model (Zheng et al. 2022). Candidate variants (SNPs and SVs) were retained only if they were exclusively present in the small‐width belted individual and absent in all 10 normal‐width belt samples.

### 2.7 Nucleotide Diversity Analysis

To gain insight into the local diversity patterns within the CNV region compared to the rest of the genome and other breeds, we calculated the nucleotide diversity using 10 Dutch Belted samples with a normal‐width belt, as wel as with 10 Dutch Friesian Red and nine Groningen White Headed individuals. Short‐reads from these animals were aligned against the *de novo* generated Dutch Belted assembly using Minimap2 v2.28‐r1209 with the ‐ax sr preset (Li 2018). Small variants were called on these alignments using Clair3 v1.0.9 with the Illumina model (Zheng et al. 2022), and merged using BCFtools v1.22 (Danecek et al. 2021). Subsequently, nucleotide diversity was calculated using VCFtools v0.1.17 (Danecek et al. 2011) with a window size of 25 kb. Visualisation was performed in R 4.5.2 (R Core Team 2021) using ggplot2 v4.0.1 (Wickham 2016).

## 3 Results

### 3.1 Challenges in Finding the CNV in the Dutch Belted

To investigate the CNV behind the belted phenotype in detail, we first needed to confirm that our reads captured the CNV and update its genomic coordinates from the old UMD3.1 assembly (BTA3:118 578 893–118 616 348) to the current ARS‐UCD2.0 reference genome (Awasthi Mishra et al. 2017). Our initial attempt to locate the CNV in whole genome sequence data from Dutch belted individuals (10 with short‐reads, two with both long‐ and short‐reads), used multiple structural variant callers. For this purpose we sequenced 10 Dutch Belted individuals with short‐reads (Illumina PE 150) and two individuals with both long‐reads (ONT PromethION) and short‐reads. After trimming, short‐read samples achieved a mean coverage of 10.5×, while long‐read samples achieved a mean coverage of 28×. However, none of the tools detected the CNV in any sample, regardless of the sequencing technology or detection method.

We therefore turned to read coverage analysis of alignments against ARS‐UCD2.0. This revealed a 6 kb region approximately 16.5 kb upstream of *TWIST2* with increased coverage (a four‐fold increase in the short‐reads and a twofold increase in the long‐reads), consistent with previous findings (Awasthi Mishra et al. 2017; Rothammer et al. 2018). Accordingly, we annotated this region (BTA3:118 028 480–118 034 892) as the location of the CNV in the current reference cattle genome assembly, ARS‐UCD2.0.

### 3.2 Assembling the Dutch Belted Genome

To investigate the CNV further, we generated a *de novo* whole‐genome assembly from the normal‐width Dutch Belted. This assembly achieved a complete and single‐copy BUSCO score of 94.0% and genome statistics comparable to the current reference cattle genome (Table 1). Notably, our Dutch Belted assembly showed much higher contiguity (N50 = 102.8 Mb compared to N50 = 26.4 Mb for ARS‐UCD2.0). Repeat modelling identified 318 repeat families present, comprising 43.18% of the Dutch Belted genome assembly. The CNV is even more repeat rich than the rest of the genome, with 63.15% of the CNV region consisting of repeats. Although several reads spanned the entire CNV, the assembly reconstructed only three out of the four copies. This is likely due to the high similarity between the copies; in particular, copies two and three share identical breakpoints, preventing the assembler from distinguishing them. Nevertheless, the assembly is of high quality and offers a good representation of the Dutch Belted genome.

**TABLE 1:** Comparison of the *de novo* generated Dutch Belted Assembly with ARS‐UCD2.0. BUSCO scores consist of the following: Complete and single‐copy (S), complete and duplicated (D), fragmented (F), and missing (M), all in percentages of the total.

Metric	Dutch Belted	ARS‐UCD2.0
# Contigs	2727	2344
Total length	2 752 313 723	2 770 686 120
Largest contig	157 537 324	158 534 110
N50	102 811 481	26 402 946
L50	12	32
BUSCO	94.0 (S), 1.8 (D), 1.2 (F), 3.0 (M)	97.6 (S), 1.2 (D), 0.5 (F), 0.8 (M)

*Note:* Study data.

### 3.3 Effect of Copy Number Variation on Belted Phenotypes

To examine potential associations between CNV copy number and abnormal belt phenotypes, we analysed samples from cattle exhibiting normal‐width belts versus those with reduced belt width and those with an incomplete belt. Copy number was estimated using three complementary approaches: coverage‐based short‐read sequencing, qPCR, and haplotype‐resolved long‐read sequencing using Oxford Nanopore Technologies (ONT).

To address limitations in Rothammer et al. (2018), where low DNA concentrations (< 10 pg/μL) hindered differentiation between homozygous and heterozygous belted animals, we performed a similar qPCR analysis with a final DNA concentration of 644 pg/μL in the reaction mixture, approximately 64‐fold higher than their minimum concentration (Table 2). Using the Comparative Ct (ΔΔCt) method, we quantified CNV copy number via qPCR in six atypical belted samples (three small‐width belted and three incomplete‐belted) and one non‐belted control. For each sample, Ct values from triplicate wells were averaged, and ΔΔCt was calculated relative to the non‐belted control (assumed copy number of two). Copy number was inferred as $2\times 2^{-\Delta \Delta C_{t}}$. Among the small‐width belted samples, qPCR revealed a mean of 8.0 ± 1.7 copies (seven copies in two individuals and 10 copies in the remaining individual). Among the incomplete‐belted samples, qPCR revealed a mean of 5.0 ± 1.4 copies (four copies for one individual and six copies for the other one). The control sample showed the expected two copies.

**TABLE 2:** The number of CNV copies found in each sample of this study, as well as their phenotype and the method by which the number of copies was derived. Haplotype‐level results (H1 and H2) were derived from Oxford Nanopore Technologies (ONT) long‐read sequencing and analysed using BLAST to count CNV copies per haplotype. A normal‐width belt refers to a belt that is complicit with the Dutch Belted studbook, a small‐width belt is one that is less than 25 cm at its thinnest point, and an incomplete belt is one that does not completely envelop the midsection. The coverage method consists of using the increase of read coverage in the CNV region compared to the rest of BTA3. The control sample was taken from a non‐Dutch belted individual without a belt.

Sample ID	No copies within the CNV	Phenotype	Method	Sequencing technology
NormB1	8	Normal‐width belt	Coverage	Illumina
NormB2	8	Normal‐width belt	Coverage	Illumina
NormB3	8	Normal‐width belt	Coverage	Illumina
NormB4	7	Normal‐width belt	Coverage	Illumina
NormB5	7	Normal‐width belt	Coverage	Illumina
NormB6	8	Normal‐width belt	Coverage	Illumina
NormB7	5	Normal‐width belt	Coverage	Illumina
NormB8	8	Normal‐width belt	Coverage	Illumina
NormB9	8	Normal‐width belt	Coverage	Illumina
NormB10	8	Normal‐width belt	Coverage	Illumina
NormB11	11	Normal‐width belt	Coverage	Illumina
NormB12	7	Normal‐width belt	Coverage	Illumina
NormB12H1	4	Normal‐width belt	BLAST	ONT
NormB12H2	4	Normal‐width belt	BLAST	ONT
SmallB1	10	Small‐width belt	Coverage	Illumina
SmallB1H1	4	Small‐width belt	BLAST	ONT
SmallB1H2	4	Small‐width belt	BLAST	ONT
SmallB2	10	Small‐width belt	qPCR	—
SmallB3	7	Small‐width belt	qPCR	—
SmallB4	7	Small‐width belt	qPCR	—
IncomB1	6	Incomplete belt	qPCR	—
IncomB2	6	Incomplete belt	qPCR	—
IncomB3	4	Incomplete belt	qPCR	—
IncomB4H1	4	Incomplete belt	BLAST	ONT
IncomB4H2	—	Incomplete belt	BLAST	ONT
IncomB5H1	4	Incomplete belt	BLAST	ONT
IncomB5H2	—	Incomplete belt	BLAST	ONT
IncomB6H1	4	Incomplete belt	BLAST	ONT
IncomB6H2	—	Incomplete belt	BLAST	ONT
Control	2	No belt	qPCR	—

*Note:* Study data. H1 and H2 denote haplotypes from long‐read sequencing. For incomplete belt samples (IncomB4H2, IncomB5H2, IncomB6H2), ‘–’ indicates that reads did not fully capture both strands of the CNV, preventing conclusive copy number estimation for that haplotype.

For normal‐width belted samples, coverage‐based estimates ranged from mean = 8.0 ± 1.3 (5 to 11 copies; Table 2). The high overlap between groups suggests that copy number alone does not determine belt width, aligning with previous research (Awasthi Mishra et al. 2017; Rothammer et al. 2018).

To resolve whether the overlap in total copy number reflected uniform haplotype composition, we used ONT long‐reads spanning the entire CNV region, enabling all copies within a single haplotype to be captured. Analysis of long‐reads from one normal‐width belted individual, one small‐width belted individual, and three individuals with incomplete belts revealed that all resolvable haplotypes contained four CNV copies (Table 2). For incomplete belt samples, only one haplotype per individual (H1) could be conclusively resolved due to insufficient coverage of both strands; the second haplotype (H2) lacked reads that completely captured the CNV. No evidence was found for haplotypes containing more or fewer than four copies, suggesting that four‐copy haplotypes may be predominant in the Dutch Belted population, regardless of belt completeness.

Together, coverage‐based estimates, qPCR results, and haplotype‐resolved long‐read data consistently show substantial overlap in CNV copy number across phenotypic groups, with no haplotype‐level differentiation detected between complete and incomplete belt phenotypes. These results suggest that CNV copy number alone does not explain variation in belt width or completeness (Awasthi Mishra et al. 2017; Rothammer et al. 2018).

### 3.4 Width Related Variations

The width of the belt is a defining trait in Dutch Belted cattle, but the genetic basis of this variation remains unclear. To explore whether variation in belt width could be explained by genetic differences, we compared a small‐width belted individual to the normal‐width *de novo* assembly.

We used long‐ and short‐reads of a Dutch Belted with a small‐width belt to find potential width‐related genetic variants, and utilised variant calling to compare these reads against the Dutch Belted *de novo* assembly. Long‐reads were used to identify structural variants (insertions, deletions, duplications, and inversions of > 50 bp), while small variants, SNPs and indels (<= 50 bp), were identified using short‐reads. SNP and SV candidate variants were only retained if they were exclusively present in the small‐width belted individual and absent in all 10 normal‐width belt samples. This yielded 201 669 SNPs and 79 938 indels unique to the small‐width belted animal; however, none of these variants were located within the CNV or the *TWIST2* locus and its surrounding region, which is known to (in)directly influence melanocyte development (Awasthi Mishra et al. 2017; Dai et al. 2024; Erickson et al. 2023). Moreover, no large‐scale variants, such as CNVs, insertions or inversions, were detected across the genome. Thus, while thousands of small variants remain, it is impossible to distinguish which variant is potentially causal for the width phenotype.

### 3.5 CNV Nucleotide Diversity

As shown above, no unique variants were detected within the CNV in the small‐width belted sample. This suggests that, with fewer than 12 individuals, all variation within the CNV was already captured, indicating very low genetic diversity in this region.

**FIGURE 1:**
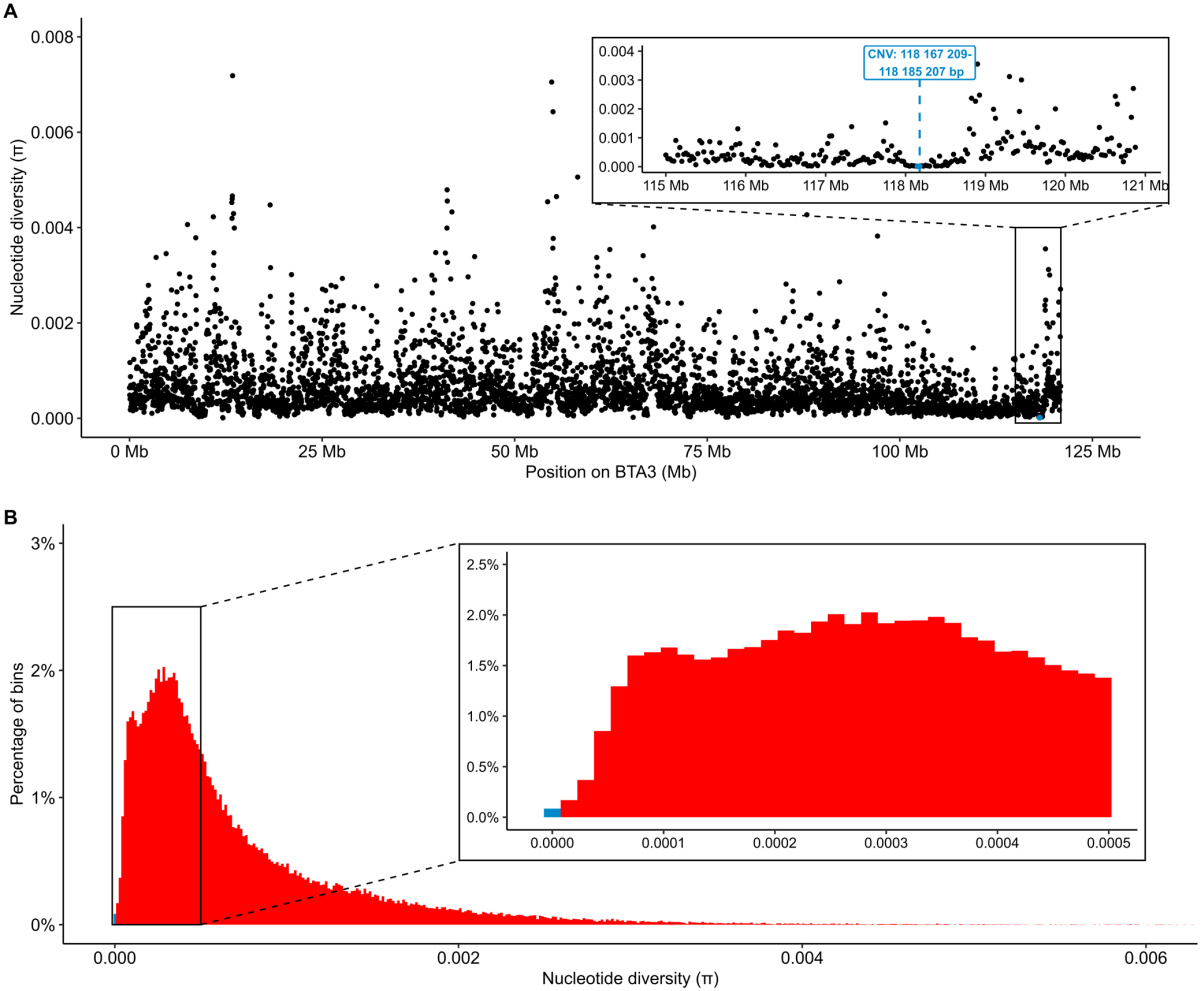
Nucleotide diversity across BTA3 in Dutch Belted cattle. (A) Nucleotide diversity (π) across the entire length of BTA3, calculated in 25 kb non‐overlapping windows from short‐read sequencing data of 11 Dutch Belted individuals. The location of the belted‐copy‐number‐variant (CNV; BTA3:118 167 209–118 185 207) is indicated by the blue dashed line, and the overlapping genomic windows are highlighted with blue data points. The inset shows a magnified view of the genomic region surrounding the CNV. (B) Distribution of nucleotide diversity values for all 25 kb windows on BTA3. The blue bar represents the windows containing the belted CNV, illustrating its position relative to chromosome‐wide nucleotide diversity.

To assess nucleotide diversity within the CNV systematically, we compared the number of variants inside the CNV with those outside using a window size of 25 kb Figure 1. For this, we used short‐read data from 11 Dutch Belted individuals with normal‐width belts. Examining BTA3 revealed substantial variation in nucleotide diversity along the chromosome (Figure 1A). Within this context, the CNV lies in a region characterised by strikingly low diversity and appears positioned at the boundary of a likely recent selective sweep. Genome‐wide binning confirmed that the low diversity found in the CNV is rare (0.04th percentile of bins) (Figure 1B). A comparison of nucleotide diversity distributions revealed that BTA3's diversity profile closely mirrors the genome‐wide pattern (Figure S3), with only minor chromosome‐specific shifts.

To contextualise this pattern, we performed the same analysis with short‐reads from 10 Dutch Friesian Red individuals as well as with nine Groningen White Headed cattle (Figures S1 and S2). While the diversity in the Groningen White Headed is relatively low (2.85th percentile), this is still a lot higher than the near absence of diversity found in the Dutch Belted. The Dutch Friesian Red, on the other hand, shows a much higher amount of nucleotide diversity (29.4th percentile), showing that this region is not devoid of diversity in the entirety of the Dutch cattle landscape, but instead localised in the Dutch Belted. When zooming out it also becomes apparent that it is not just the CNV itself that suffers from low nucleotide diversity; up to around 20 Mb upstream of the CNV the chromosome is less diverse than these same regions in the other breeds. Taken together, these results indicate a recent reduction in genetic variation, probably driven by strong artificial selection for the belted phenotype.

## 4 Discussion

In accordance with previous research, the individuals in our dataset contained mostly seven or eight copies of the CNV, likely corresponding to *3/4* and *4/4* copy number genotypes (Awasthi Mishra et al. 2017; Rothammer et al. 2018). Coverage‐based and qPCR estimates showed that normal‐width and small‐width belted animals overlapped in their total CNV copy number ranges, indicating no association between copy number and reduced belt width. For the incomplete‐belted animals, qPCR suggested a somewhat lower total copy number (four to six copies). However, long‐read sequencing provided a different perspective; all long‐read–sequenced individuals, normal‐width, small‐width belted, and incomplete‐belted, carried at least one haplotype with exactly four copies of the CNV, and no haplotypes with two, three, or more than four copies were observed. These results indicate that the four‐copy CNV haplotype is necessary for belt formation but insufficient to explain variation in belt width or completeness, implying that additional genetic factors, possibly at unlinked loci, also influence these traits. Comparable cases have been described in pigs, where belt width and completeness depend on modifiers distinct from the primary *KIT* belt locus. For instance, variation at *MC1R*, which affects the background eumelanin–phaeomelanin balance, and allelic differences at *EDNRB*, involved in melanocyte migration, could conceivably modify the visual extent and completeness of the white belt by altering pigment distribution and contrast with surrounding areas (Giovannini et al. 2023; Schiavo et al. 2020).

Despite analysing over 200 000 SNPs and nearly 80 000 indels unique to a small‐width belted animal, none were located within the CNV or *TWIST2*. However, with data from only one small‐width belted individual, we cannot conclude that width‐related variants are absent from these regions in the broader population. Coupled with the lack of a clear copy number effect on belt width, this suggests that modifiers influencing belt width may lie outside the CNV region, or involve subtle regulatory mechanisms undetectable in our dataset. To test this possibility rigorously, larger cohorts of small‐width belted and incomplete‐belted individuals will be essential, ideally with long‐read sequencing to detect structural variants potentially missed here.

In summary, while the CNV near *TWIST2* is clearly required for belt formation, its copy number does not seem to dictate belt width or completeness. Resolving the full structure of this CNV and identifying additional modifier loci will require larger datasets and improved sequencing strategies, such as utilising the newest generation of ONT sequencing and higher coverage sequencing. From both a scientific and breeding perspective, these efforts are essential to fully understand the genetic architecture of the belted phenotype and to balance trait selection with the maintenance of genetic diversity in the Dutch Belted population.

Large structural variants are notoriously difficult to detect, and the CNV underlying the belted phenotype was missed by standard variant callers (MacDonald et al. 2014), being identifiable only through visual inspection of aligned reads, which is impractical for large‐scale studies. It is known that variant callers perform better for deletions than insertions (Teo et al. 2012), with similar biases reported for CNVs in cattle (Ayalew et al. 2024), American mink (Davoudi et al. 2022), and humans (Zhou et al. 2018). Detection is further complicated by the repeat‐rich CNV region (two‐thirds repetitive), which reduces mapping accuracy. Even the *de novo* assembly resolved only three of four copies, likely due to breakpoint ambiguity. Long reads spanning the CNV helped, but fully spanning reads were limited, so detection remained challenging even with combined short‐ and long‐read data.

Finally, our population‐level analysis revealed that the CNV region exhibits extremely low nucleotide diversity, among the lowest genome‐wide in Dutch Belted cattle, and this reduction is breed‐specific. In Dutch Friesian Red and Groningen White Headed individuals, diversity in this region fell within the 29.4th and 2.85th percentiles, substantially higher than the 0.04th percentile in Dutch Belted. This contrast indicates that the depletion of variation reflects a recent, lineage‐specific event rather than an inherent feature of the locus.

This pattern is consistent with a selective sweep, likely driven by the strong artificial selection that breeders exhibit due to their preference for the belt, amplified by the small effective population size and strict studbook. A CNV embedded within a region of such low diversity raises several biological and population‐genetic considerations. The reduction in variation is unlikely to reflect purifying selection on the CNV sequence itself, as a large proportion of the CNV consists of repetitive elements with no clear functional constraint. This reduces the plausibility that the CNV is inherently conserved for functional reasons. Furthermore, because long‐read sequencing showed that all belted animals share a similar four‐copy haplotype structure, the observed sweep potentially acted on a single ancestral CNV haplotype, which subsequently rose to high frequency through breeder‐mediated selection. Similar patterns have been reported for pigmentation traits in other domestic species, where a single derived allele becomes effectively fixed within a closed breeding population (Andersson 2012; Campagna et al. 2022; Ramey et al. 2013).

## 5 Conclusion

Our study refines the understanding of the genetic architecture underlying the belted phenotypes in Dutch Belted cattle. We confirm that the CNV upstream of *TWIST2* is necessary for belt formation, but total CNV copy number alone does not explain variation in belt width or completeness. Instead, we hypothesise that additional modifier loci elsewhere in the genome likely influence belt width and belt completeness.

Importantly, we found no evidence for width‐determining variants within the CNV or *TWIST2*, suggesting that other genomic elements must be responsible for this breeder‐relevant trait. The CNV region showed extremely low nucleotide diversity within the Dutch Belted, consistent with a signature of selection driven by strong artificial selection in a closed population. The pattern, thus, indicates a strong selective sweep in the belted population, where a single CNV haplotype was selectively propagated.

Future work should focus on haplotype‐resolved long‐read sequencing to fully characterise CNV architecture, and on larger cohorts to identify potential modifiers of belt width. Such insights will not only advance our understanding of pigmentation genetics in cattle but also support the development of genetic tools for breeders seeking to balance selection for the belted phenotype with the maintenance of genetic diversity.

## Author Contributions

Aniek C. Bouwman and Richard P.M.A. Crooijmans conceived the study. Mirte Bosse, Aniek C. Bouwman, and Martien A.M. Groenen led the project. Rensco A.H. Hogers wrote the manuscript and analysed the majority of the data. Rayner González‐Prendes conducted and analysed part of the Effect of copy number variation on belted phenotypes experiment. Kimberley Laport conducted data generation and experiments. All authors read and approved the final manuscript.

## Funding

The ReDiverse project (and its funders: the Netherlands Organisation for Scientific Research (NWO) under (Grant ALWSA.2016.5), and the European Union's Horizon 2020 research and innovation programme under grant agreement no. 696231) is acknowledged for (funding the) generation of the sequencing data. This research was funded by the Wageningen University & Research Knowledge Base Programme KB34 “Circular & Climate Neutral Society” – Project KB‐34‐013‐002, that is supported by financing from the Netherlands' Ministry of Agriculture, Fisheries, Food security and Nature (LVVN).

## Conflicts of Interest

The authors declare no conflicts of interest.

## Supporting information


**Figure S1:** Nucleotide diversity across BTA3 in Dutch Friesian Red cattle. (A) Nucleotide diversity (π) across the entire length of BTA3, calculated in 25 kb windows from short‐read sequencing data of 10 Dutch Friesian Red individuals. The location of the belted copy‐number variant (CNV; BTA3:118 167 209–118 185 207) is indicated by a blue dashed line, and the overlapping genomic windows are highlighted with blue data points. The inset shows a magnified view of the genomic region surrounding the CNV. (B) Distribution of nucleotide diversity values for all 25 kb windows across the genome. The blue bar highlights the bins corresponding to the belted CNV, illustrating its position relative to background nucleotide diversity.
**Figure S2:** Nucleotide diversity across BTA3 in Groningen White Headed cattle. (A) Nucleotide diversity (π) across the entire length of BTA3, calculated in 25 kb windows from short‐read sequencing data of nine Groningen White Headed individuals. The location of the belted copy‐number variant (CNV; BTA3:118 167 209–118 185 207) is indicated by a blue dashed line, and the overlapping genomic windows are highlighted with blue data points. The inset shows a magnified view of the genomic region surrounding the CNV. (B) Distribution of nucleotide diversity values for all 25 kb windows across the genome. The blue bar highlights the bins corresponding to the belted CNV, illustrating its position relative to background nucleotide diversity.
**Figure S3:** Distribution of nucleotide diversity (π) in Dutch Belted cattle, generated using a window size of 25 kb. Density plots comparing nucleotide diversity across the genome (red) and BTA3 specifically (teal). The similar shapes of both distributions indicate that BTA3's diversity profile is representative of the broader genomic landscape, with minor shifts reflecting chromosome‐specific variation.

## Data Availability

Genomic data, including the Dutch Belted assembly and the short‐ and long‐reads, are available at the European Nucleotide Archive under project accession PRJEB106319.
