# Supplementary material for: Untangling the Copy Number Variation at the Basis of Belted Phenotypes in Cattle Using Long‐Read Sequencing
Source: Anim Genet. 2026 Jul 17;57(4):e70159. doi: 10.1002/age.70159 (PMC13377943; doi:10.1002/age.70159)
Supplement: Supplementary file 1 — Figure S1: Nucleotide diversity across BTA3 in Dutch Friesian Red cattle. (A) Nucleotide diversity (π) across the entire length of BTA3, calculated in 25 kb windows from short‐read sequencing data of 10 Dutch Friesian Red individuals. The location of the belted copy‐number variant (CNV; BTA3:118 167 209–118 185 207) is indicated by a blue dashed line, and the overlapping genomic windows are highlighted with blue data points. The inset shows a magnified view of the genomic region surrounding the CNV. (B) Distribution of nucleotide diversity values for all 25 kb windows across the genome. The blue bar highlights the bins corresponding to the belted CNV, illustrating its position relative to background nucleotide diversity. Figure S2: Nucleotide diversity across BTA3 in Groningen White Headed cattle. (A) Nucleotide diversity (π) across the entire length of BTA3, calculated in 25 kb windows from short‐read sequencing data of nine Groningen White Headed individuals. The location of the belted copy‐number variant (CNV; BTA3:118 167 209–118 185 207) is indicated by a blue dashed line, and the overlapping genomic windows are highlighted with blue data points. The inset shows a magnified view of the genomic region surrounding the CNV. (B) Distribution of nucleotide diversity values for all 25 kb windows across the genome. The blue bar highlights the bins corresponding to the belted CNV, illustrating its position relative to background nucleotide diversity. Figure S3: Distribution of nucleotide diversity (π) in Dutch Belted cattle, generated using a window size of 25 kb. Density plots comparing nucleotide diversity across the genome (red) and BTA3 specifically (teal). The similar shapes of both distributions indicate that BTA3's diversity profile is representative of the broader genomic landscape, with minor shifts reflecting chromosome‐specific variation. [file AGE-57-0-s001.pdf]

□

APPENDIX

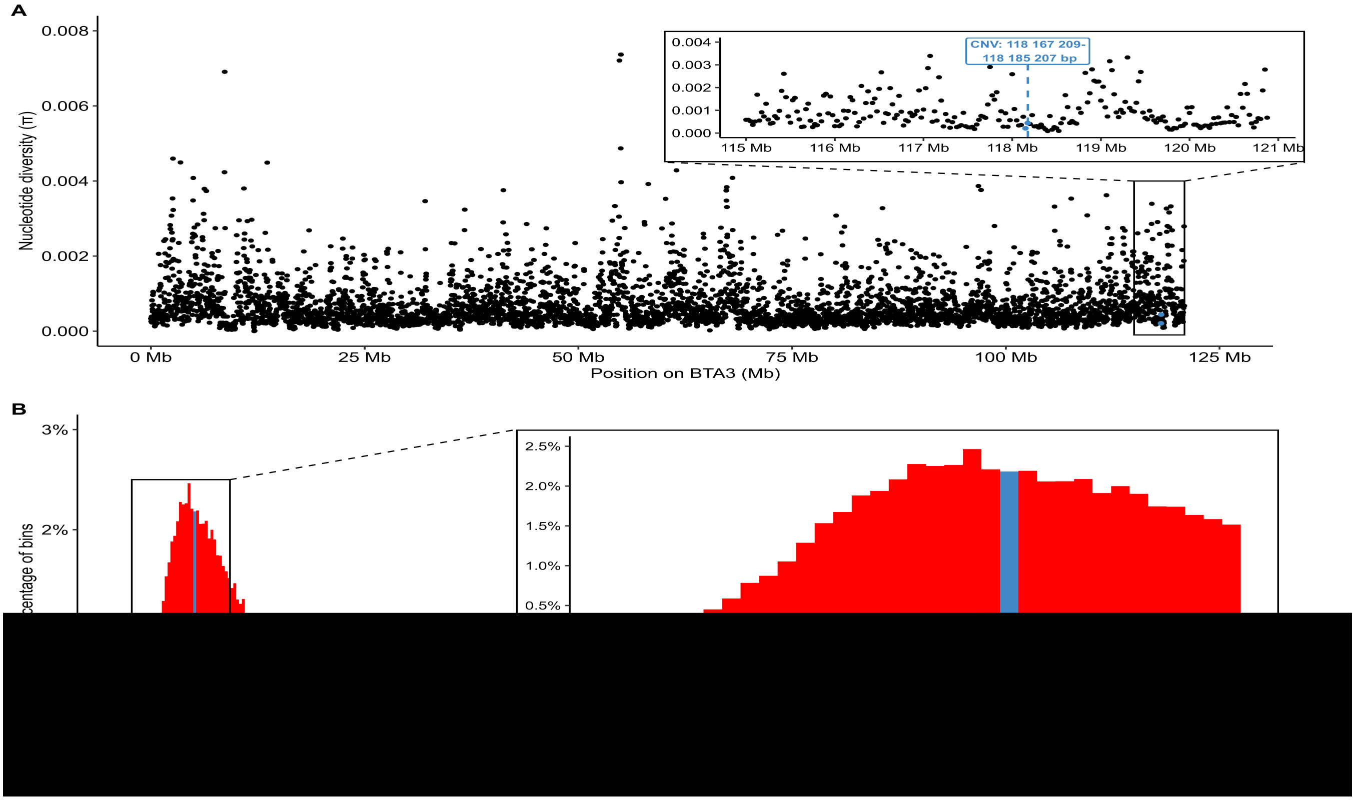

FIGURE S1 Nucleotide diversity across BTA3 in Dutch Friesian Red cattle. (A) Nucleotide diversity (π) across the entire length of BTA3, calculated in 25 kb windows from short-read sequencing data of ten Dutch Friesian Red individuals. The location of the belted copy-number variant (CNV; BTA3:118 167 209–118 185 207) is indicated by a blue dashed line, and the overlapping genomic windows are highlighted with blue data points. The inset shows a magnified view of the genomic region surrounding the CNV. **(B)** Distribution of nucleotide diversity values for all 25 kb windows across the genome. The blue bar highlights the bins corresponding to the belted CNV, illustrating its position relative to background nucleotide diversity.

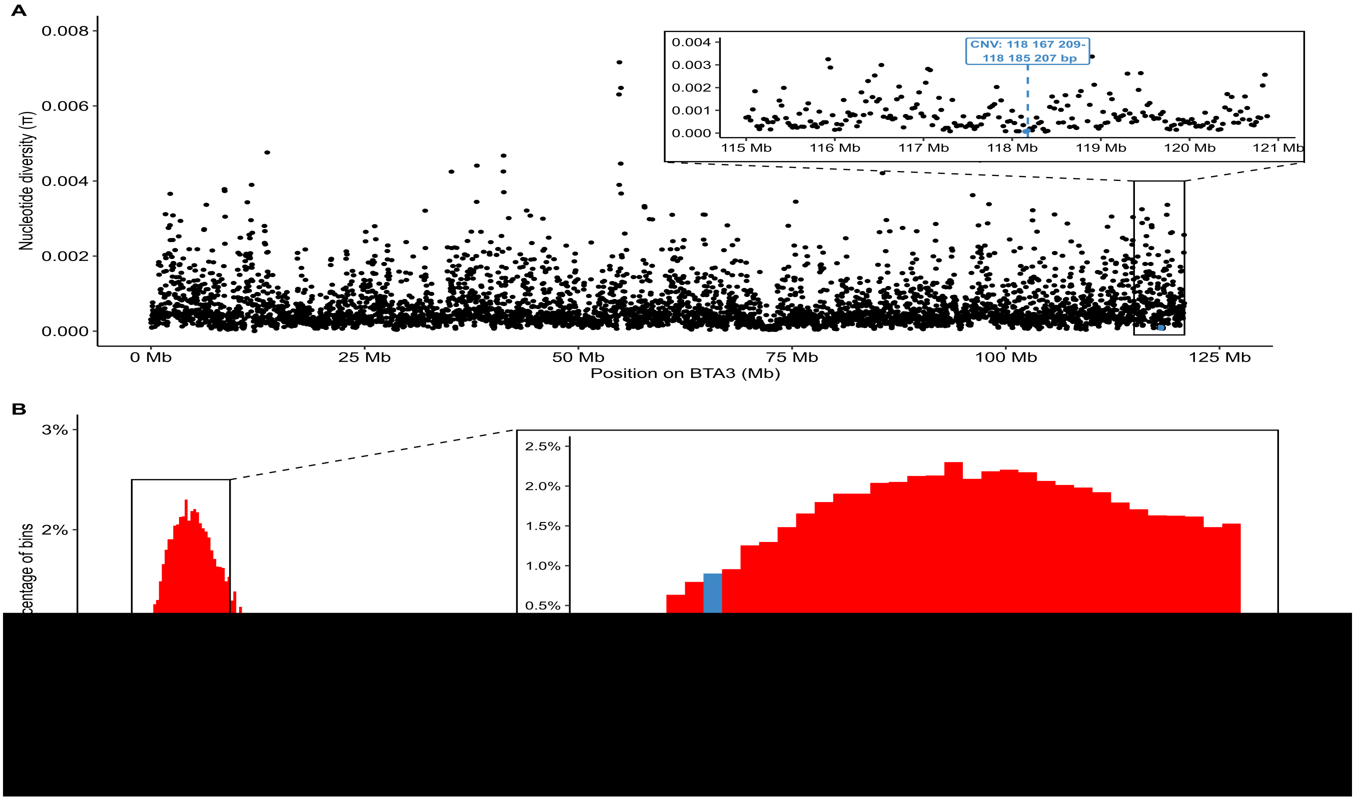

[redacted]

FIGURE S2 Nucleotide diversity across BTA3 in Groningen White Headed cattle. **(A)** Nucleotide diversity (π) across the entire length of BTA3, calculated in 25 kb windows from short-read sequencing data of nine Groningen White Headed individuals. The location of the belted copy-number variant (CNV; BTA3:118 167 209–118 185 207) is indicated by a blue dashed line, and the overlapping genomic windows are highlighted with blue data points. The inset shows a magnified view of the genomic region surrounding the CNV. **(B)** Distribution of nucleotide diversity values for all 25 kb windows across the genome. The blue bar highlights the bins corresponding to the belted CNV, illustrating its position relative to background nucleotide diversity.

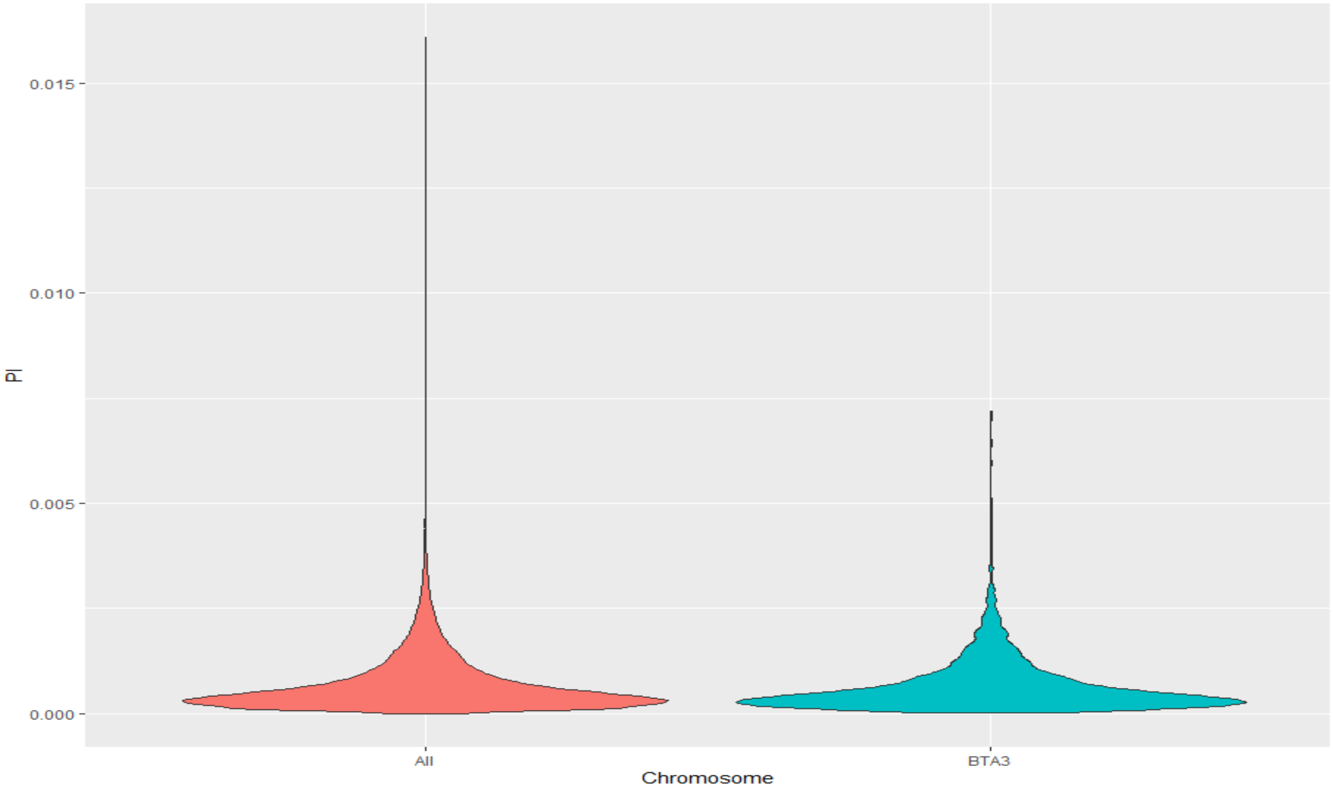

FIGURE S3 Distribution of nucleotide diversity (π) in Dutch Belted cattle, generated using a window size of 25 kb. Density plots comparing nucleotide diversity across the genome (red) and BTA3 specifically (teal). The similar shapes of both distributions indicate that BTA3's diversity profile is representative of the broader genomic landscape, with minor shifts reflecting chromosome-specific variation.
